# Editorial

**Published:** 1860-07

**Authors:** 


### EDITORIAL.

### AMERICAN MEDICAL ASSOCIATION.

From the reports in the New Haven papers, and our own notes, we have been able to give a more full and correct account of the doings of the recent annual meeting of the American Medical Association, than we have seen in any of our exchanges. The meeting was very fully attended, the number of members registered being over six hundred. The most cordial good feeling was exhibited throughout the session. All who listened to the reading of papers and the discussions in the several sections, were very much pleased with the practical working of that feature of the recent meeting. The profession and citizens of New Haven left nothing undone that was calculated to add to the interests or pleasure of the members of the Association.

The numerous evening parties were extremely pleasant, and the general entertainment in the State House on Thursday evening, was truly a grand social gathering, and a season of gay festivity. If any failed to enjoy themselves, the fault must have been in their own minds, for smiling faces, beautiful flowers, delicious strawberries met them at every turn. At the close of the session, the opinion seemed to be expressed, with great unanimity, that it had been more profitable and satisfactory than any other since the organization of the Association.

It will be seen by the record of proceedings, that the next annual meeting is to be held in this city on the 1st Tuesday in June, 1861. And we have no doubt but our citizens as well as the profession of the city and State, will receive the members of the Association on that occasion, with a cordiality that has not been excelled in any other part of the Union.

### CONVENTION OF DELEGATES FROM MEDICAL COLLEGES.

According to the adjournment at Louisville, the Delegates from Medical Colleges, met in New Haven on Monday, preceding the day for the meeting of the American Medical Association.

The official record of the proceedings is given in the present number of the Journal. Although that record is strictly correct, and contains all the propositions acted upon, and the results of such action ; yet it gives but an imperfect idea of the sentiments expressed by those who participated in the proceedings. Some allegations were made, which, if true, ought to be known to the whole profession ; and if not true, the parties implicated should promptly disprove them.

For instance, when the proposition recommending the Colleges to rigidly exact reliable evidence that every candidate for graduation had actually studied medicine *three* full years, and *attended* two courses of lectures, it was alleged that some of the oldest, largest, and most influential schools in the Union, habitually disregard this rule ; and annually graduate students who have, not only, not studied the *three* years, but who have not *attended* even one third of the course of lectures at the close of which they graduate. Such violations of the plainest rules of propriety, as well as the most important dictates of justice, both to the profession and the community, cannot be too strongly censured. And more especially when they are practised by colleges long established, and the halls of which are annually overflowing with large classes.

Another noticeable feature of that convention was the absence of all delegates from the schools here referred to.

Do the faculties of such schools think that all the complaints of the profession in regard to medical education are really groundless and unworthy of notice? Or have they become so secure in their prosperity that they think they are beyond the reach of criticism? In either case the time may come when they will both feel and heed the sentiments of the profession as well as the interests of humanity.

---

Dr. William Pepper, formerly a clinical teacher in the Pennsylvania Hospital, and one of the most eminent practitioners of Philadelphia, has been appointed Professor of the Theory and Practice of Medicine in the University of Pennsylvania, vice Dr. Wood, resigned.

### MEDICAL COLLEGES 1859-60.

We take the following statistics chiefly from the *American Medical Gazette*:—

| | Students. | Graduates. |
|---|---|---|
| Jefferson Medical College, Phila., | 630 | 170 |
| University of Pennsylvania, Phila., | 515 | 173 |
| University of Nashville, Nashville, Tenn., | 456 | 101 |
| University of New York, | 411 | 138 |
| Medical College of South Carolina, Charleston, | 248 | |
| College of Physicians, New York City, | 200 | 55 |
| Massachusetts Medical College, Boston, | 195 | 32 |
| Atlanta Medical College, Atlanta, Ga., | 166 | 50 |
| University of Louisville, Ky., | 130 | 38 |
| Kentucky School of Medicine, Louisville, Ky. | | 37 |
| Ohio Medical College, Cincinnati, | 123 | 31 |
| Rush Medical College, Chicago, Ill., | 101 | 36 |
| Cincinnati College of Medicine and Surgery, | 97 | 30 |
| Cleveland Medical College, Cleveland, Ohio, | 70 | 18 |
| New York Medical College, | 75 | 20 |
| Buffalo Medical College, Buffalo, N. Y., | 70 | |
| Oglethorpe Medical College, Savannah, Ga., | 60 | 21 |
| Savannah Medical College, " | | 14 |
| Medical Department of Yale College, | | 13 |
| Lind University, Chicago, Ill., | 30 | 9 |
| Med. Dep. of the Pennsylvania College, Phila., | | 14 |
| Mobile Medical College, Mobile, Ala., | 50 | 15 |
| College of Pharmacy, Phila., | | 38 |
| Shelby Medical College, Nashville, Tenn., | 65 | 9 |
| University of Louisiana, New Orleans, | 401 | 113 |
| New Orleans School of Medicine, | 216 | 63 |
| Medical Department of Iowa University, | 80 | 42 |
| St. Louis Medical College, | | 52 |
| Missouri Medical College, St. Louis, | | 30 |
| Medical College of Alabama, | | 15 |
| Medical College of Virginia, Richmond, | | 82 |
| Medical College of Georgia, | | 62 |
| Pennsylvania Medical College, Phila., | | 38 |
| National Medical College, Washington, D. C., | 83 | 29 |

### NOTICE TO MEDICAL MEN IN ILLINOIS.

The undersigned were appointed at the last meeting of the Illinois State Medical Society, a Committee on Drugs and Medicines.

In the constitution of the Society, we find the duties of the committee to be as follows:

"The committee on Drugs and Medicines shall prepare an annual report on all improvements and discoveries in Pharmacy and on Materia Medica effected during the year, and on all subjects connected with the sophistication and sale of Drugs and Medicines."

The committee would respectfully request the physicians of this State to report in reference to this subject, the result of their observations, and send as soon as the 1st of April 1861, to:

F. K. BAILEY, *Chair'n*, Joliet,
R. G. LAUGHLIN, Heyworth, McLean Co.
Or, H. R. PAYNE, Marshall, Clark Co.

Joliet, July, 1860.

---

Decatur, June 9th, 1860.

Dr. N. S. Davis,

Dear Sir:—

Please give the following a place in the *Examiner*, and much oblige

Your Friend,
S. T. TROWBRIDGE.

---

### TO THE MEDICAL PROFESSION OF ILLINOIS.

The committee on Practical Medicine appointed at the last sitting of the Illinois State Medical Society, would respectfully invite the co-operation of its members, and the profession of the State in general, in furnishing material for their report; hoping each one will feel himself individually called upon to forward to the chairman of this committee, such observations of a practical character pertaining to disease, of either epidemic, sporadic, or other nature upon which he may feel inclined to treat. We ask of each to give his own views in his own way, upon whatever subject would legitimately take a place in this report, and that the same may be submitted to us at as early a day as the 1st of February 1861. With much anxiety,

I remain your most humble Servant,

S. T. TROWBRIDGE, *Chair'n.*

---

Prof. G. B. Wood, having retired from active professional life, and being on the eve of sailing for Europe, was entertained at a public dinner by the members of the profession in Philadelphia, and the proceedings of the day were distinguished for their harmony and success.

---

*Hydrocyanate of Iron in Epilepsy.*—Dr. Trent, of Richmond, Va., has treated successfully several cases of epilepsy with the following mixture:—

℞ Hydrocyanat. Ferri, ʒi.

Pulv. Valerian, ʒij.

Sig. Prepare a mass and divide into 120 pills, of which give one pill three times a day, gradually increasing to four a day. —*Nashville Journal.*

---

*Medicine in China.*—Perfect free trade in physic exists there. The field is open to every one without examination. It is thought that every one has sense enough to choose his own doctor, and choose a good one, and if they suffer it is their own fault and they are to blame. When a physician is consulted he lays the hand on a soft cushion, feels the pulse of the wrist, asks age and symptoms, looks the patient in the face, strokes his beard, and writes the prescription perhaps, "150 pills twice a day." "A dose of Chinese medicine," says the *Lancet*, "is quite a curiosity. It is about the size of half a pound of moist sugar, and consists of twenty separate packets —four or five kinds of bark, a little orange peel, some walnuts, some gentian, and half a dozen other roots, not unlike a small cake of blacking. These are all boiled together, and a good half-pint of the decoction is to be taken *quite hot* as a dose." The profession does not seem to be highly remunerated however. The lowest fee for a visit is 60 cash, about four cents, and the highest ordinary fee is 180, or twelve cents, although as much as 240, or 360 cash, eighteen and twenty-four cents, are sometimes given.—*Nashville Journal.*

---

*Malformation of the Chest.*—Dr. Wojaczek, from Vienna, who is a native of Oslavan, in Moravia, and aged about 23, is the subject of a peculiar malformation of the chest, which has been examined by eminent medical men at the different universities and medical schools of Europe. This gentleman was introduced by Dr. Alexander Simpson, and submitted himself for examination by the members of the Medico-Chirurgical Society of Edinburgh. In front, the chest presents in the middle, at its lower part, a remarkable depression or hollow, about three inches deep, and large enough to lodge the head of a child. This hollow is formed by the inflexion of the sternum downwards and backwards towards the spinal column, which it approaches so closely, that, by calculation, only about 1½ inches intervene between the lower end of the sternum and the front of the bodies of the vertebræ. There is no deficiency of the osseous or cartilaginous textures, but the cartilages of the ribs are bent backwards to join the depressed sternum and form the sides of the hollow: the skin and soft parts present nothing unusual. In consequence of this peculiar shape of the chest, the respiration is almost exclusively carried on by the diaphragm, and was first discovered by Professor Skoda and Rokitansky during an illness, in which they had occasion to examine M. Wojaczek's chest. Casts of the malformation have peen placed in the museums of the University and of the Royal College of Surgeons.—*Edinburgh Medical Journal*, June, 1860.

